# IRX5 promotes adipogenesis of hMSCs by repressing glycolysis

**DOI:** 10.1038/s41420-022-00986-7

**Published:** 2022-04-15

**Authors:** Bulin Jiang, Liyuan Huang, Tian Tian, Hongling Wu, Hantao Yao, Tyler Marmo, Fangfang Song, Cui Huang

**Affiliations:** 1 The State Key Laboratory Breeding Base of Basic Science of Stomatology (Hubei-MOST) & Key Laboratory of Oral Biomedicine Ministry of Education (KLOBM), School & Hospital of Stomatology, Wuhan University, Wuhan, China; 2 Children’s Hospital of Philadelphia, Philadelphia, PA USA

**Keywords:** Glycobiology, Stem-cell differentiation

## Abstract

Iroquois homeobox transcription factor 5 (IRX5) plays a pivotal role in extramedullary adipogenesis, but little is known about the effects of IRX5 on adipogenesis of human bone marrow-derived mesenchymal stem cells (hMSCs). In this study, we aimed to determine the effect of IRX5 on hMSCs adipogenesis. By means of qPCR analysis, we determined that IRX5 expression was elevated during adipogenic commitment of hMSCs. The biologic role of IRX5 was further investigated by employing a gain/loss-of-function strategy using an in vitro lentivirus-based system. IRX5 overexpression promoted adipogenesis whereas IRX5 knockdown reduced the adipogenic phenotype. RNA-seq and metabolomics revealed that IRX5 overexpression repressed glycolysis. Dual-luciferase assay results showed that IRX5 overexpression transcriptionally activates peroxisome proliferator-activated receptor gamma coactivator (PGC-1α). Metformin and PGC-1α inhibitor reversed IRX5-induced adipogenesis and glycolytic inhibition. Collectively, IRX5 facilitates adipogenic differentiation of hMSCs by transcriptionally regulating PGC-1α and inhibiting glycolysis, revealing a potential target to control bone marrow-derived mesenchymal stem cells (BMSCs) fate decision and bone homeostasis.

## Introduction

Although bone marrow adipose tissue (BMAT) was discovered more than a century ago, little is known about its origin and function in bone marrow (BM) [1]. BMAT is distinct from white adipose tissues (WAT), brown adipose tissues (BAT) and beige adipose tissues. It plays significant roles in bone homeostasis and whole-body energy metabolism [2]. Postnatally, BMAT originates from progenitors that are distinct from peripheral adipose tissues. BMAT is thought to be derived from BMSCs located within BM stroma, which can give rise to osteoblasts and adipocytes [3–7]. Increasing evidence suggests that there is a theoretical inverse relationship between adipogenic and osteogenic differentiation of BMSCs both in vitro and in vivo [8, 9]. It has been reported that abnormal accumulation of adipocytes in the BM in osteoporosis, estrogen deficiency, anorexia nervosa, chronic glucocorticoid treatment, and Cushing disease leads to decreased bone mass and abnormal bone remodeling [10–13]. A clear understanding of the cellular and molecular mechanisms of BMSCs adipogenic commitment is profoundly significant for the purposes of illuminating the pathogenesis of bone and metabolic diseases and finding novel, effective therapeutic targets.

IRX5 is a family member of Iroquois homeobox transcription factors (IRX) which shares a highly conserved homeodomain from worm to vertebrates [14]. It plays an essential role in many physiological and pathological processes by binding specific sequence sites *ACANNTGT*. A causal cis-regulatory variant in the first intron of the *FTO* gene, which has been reported to be most associated with obesity, leads to activation of downstream targets IRX5 and IRX3 through long range enhancer promoter interactions. Manipulation of IRX3 and IRX5 expression in three different cellular models including mouse embryonic fibroblast–derived adipocytes, white 3T3-L1 preadipocytes, and β-adrenergic–stimulated beige ME3 preadipocytes induces adipocyte lipid accumulation and repressed thermogenesis [15]. IRX5 null mice had a significant anti-obesity phenotype associated with a dramatic loss of fat mass [16]. This evidence supports the notion that IRX5 is important for the identification and differentiation of extramedullary adipocytes. Considering the biological differences between peripheral adipose tissues and BMAT, the function of IRX5 on BMSC adipogenesis needs to be further addressed.

In this study, we showed that IRX5 overexpression promotes adipogenesis of hMSCs by increasing PGC-1α expression and inhibiting glycolysis. This effect is reversed by a glycolysis activator, metformin, and a PGC-1α inhibitor, SR-18292.

## Results

### The differentiation ability of hMSCs

We began the study by testing adipogenic and osteogenic differentiation of hMSCs. hMSCs were induced with an adipogenic medium or osteogenic medium.

**Fig. 1:**
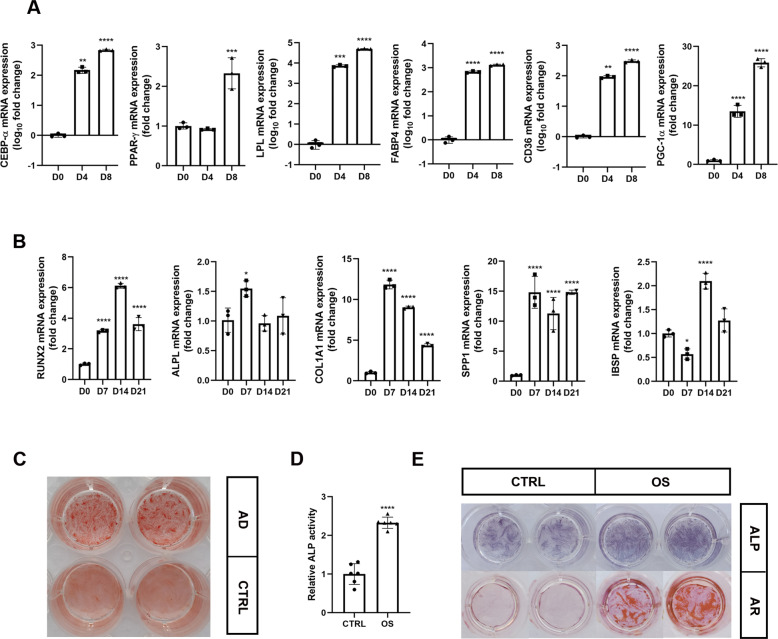
The differentiation ability of hMSCs. **A** mRNA levels of *CEBP-α*, *PPAR-γ*, *LPL*, *FABP4*, *CD36* and *PGC-1α* during hMSCs adipogenic differentiation at D0, D4 and D8 (*n* = 3). **B** mRNA levels of *RUNX2*, *ALPL*, *COL1A*, *SPP1* and *IBSP* during hMSCs osteogenic differentiation at D0, D7, D14 and D21 (*n* = 3). **C** Oil red O staining was performed at D16 after adipogenic induction. **D**, **E** ALP activity assay (*n* = 6) and ALP staining were performed at D7 (**D**, **E**), alizarin red staining was performed at D21 after osteo genic induction (**E**). Results are shown as mean ± SD. * indicates a significant difference from the control group, *p < 0.05, **p < 0.01, ***p < 0.001, ****p < 0.0001.

As expected, adipogenesis-associated genes such as *PPAR-γ*, *CEBP-α*, *LPL*, *FABP4*, *CD36* and *PGC-1α* were significantly up-regulated at D4 and D8 under adipogenic differentiation (Fig. 1A). After 16 days of adipogenic induction, Oil red O staining indicated that a substantial number of oil droplets had accumulated in cells (Fig. 1C). For osteogenic differentiation, *RUNX2*, *COL1A* and *SPP1* were up-regulated during the early stage of osteogenesis while *IBSP* was up-regulated during the later period (Fig. 1B). ALP activity assay, ALP staining, and alizarin red staining showed high ALP activity and mineralization levels in differentiated cells (Fig. 1D, E). The results therefore suggest that hMSCs is a valid cell type that can be used to study lineage differentiation.

### IRX5 overexpression and knockdown play opposite effects on adipogenic differentiation of hMSCs

**Fig. 2:**
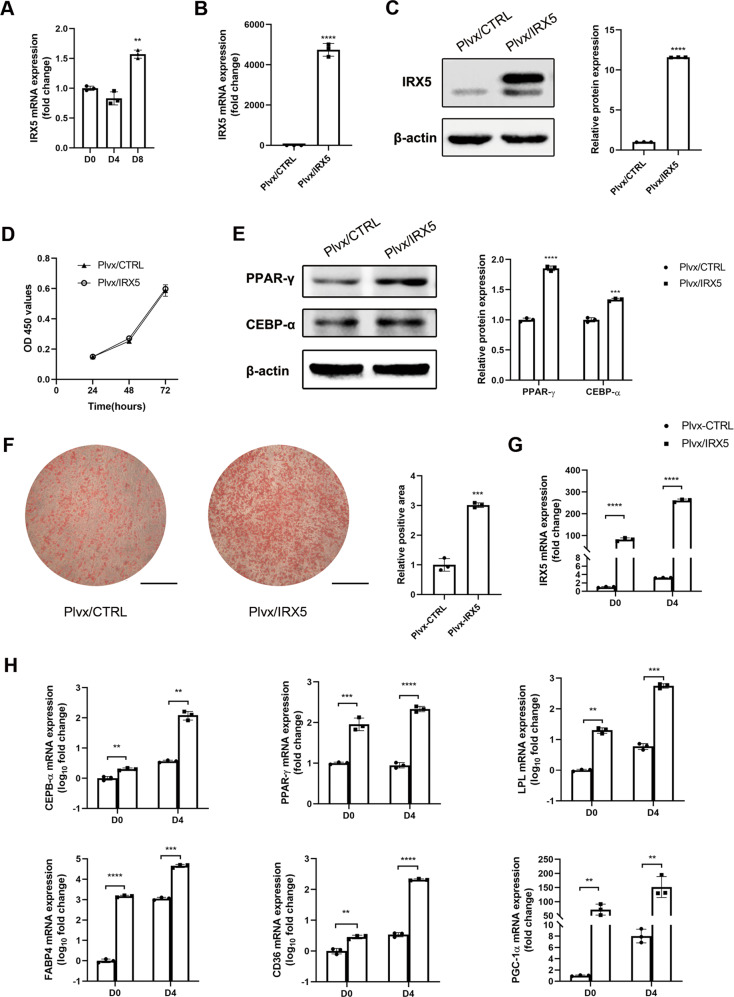
Influence of IRX5 overexpression on adipogenic differentiation of hMSCs. **A** Endogenous mRNA levels of *IRX5* during adipogenic differentiation at D0, D4 and D8. **B**, **C** IRX5 overexpression efficiency were determined by qRT-PCR (**B**) and Western blot (**C**). **D** CCK-8 assay. **E** Protein levels of PPAR-γ and CEBP-α in Plvx/CTRL and Plvx/IRX5 groups by western blot assay. **F** Oil red O staining was performed at D16 after adipogenic induction. **G** mRNA levels of *IRX5* during adipogenic differentiation at D0 and D4 in Plvx/CTRL and Plvx/IRX5 groups. **H** mRNA levels of *CEBP-α*, *PPAR-γ*, *LPL*, *FABP4*, *CD36* and *PGC-1α* during adipogenic differentiation at D0 and D4. Scale bar, 500 μm. Results are shown as mean ± SD. *n* = 3. *p < 0.05, ** *p* < 0.01, ***p < 0.001, ****p < 0.0001.

Endogenous IRX5 expression increased during adipogenic differentiation, indicating the potential role of IRX5 in regulating adipogenic differentiation (Fig. 2A).

**Fig. 3:**
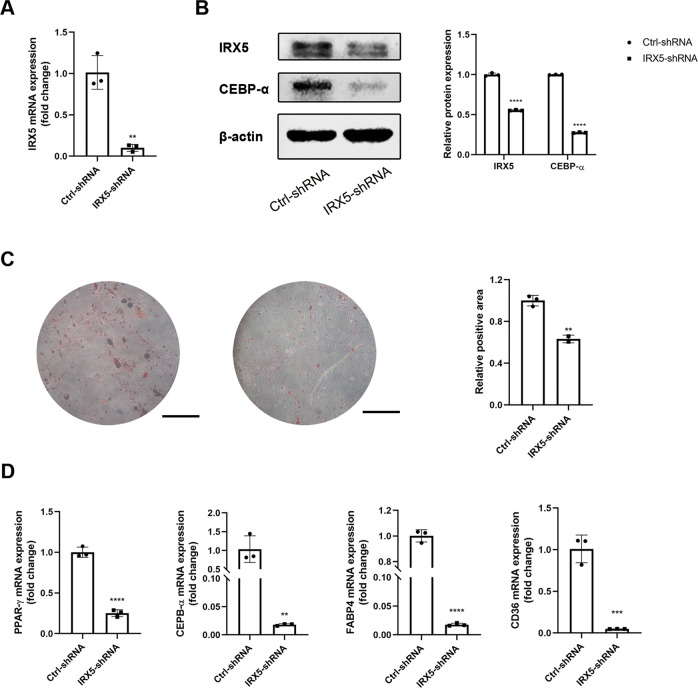
Effect of IRX5 knockdown on the adipogenic differentiation of hMSCs. **A**, **B** IRX5 knockdown efficiency were verified by qRT-PCR (**A**) and western blot (**B**). **B** IRX5 and CEBP-α protein expression. **C** Oil red O staining analysis of lipid droplets at D16 after adipogenic differentiation. **D** qRT-PCR analysis of *PPAR-γ*, *CEBP-α*, *CD36* and *FABP4* at D8 after adipogenic differentiation. Scale bar, 500 μm. Results are shown as mean ± SD. *n* = 3. * Indicates a significant difference from the control group, *p < 0.05, **p < 0.01, ***p < 0.001, ****p < 0.0001.

Overexpression efficiency was detected by qRT-PCR and western blot in hMSCs (Fig. 2B, C and Fig. S3A), in ST2 cells (Figs. S1A, B and S8A) and in HELA cell line (Figs. S2A, B and S9A). CCK-8 assay was performed to exclude the effects of IRX5 in cell proliferation. These results indicated that proliferation was not affected by infection or IRX5 overexpression (Fig. 2D). Two key transcription factors of adipogenic differentiation, PPAR-γ and CEBP-α, were upregulated in Plvx/IRX5 hMSCs as shown in western blots (Fig. 2E and Fig. S3B). Oil red O staining for lipid accumulation at day 16 showed that more lipid droplets had accumulated in the Plvx/IRX5 group than the Plvx/CTRL group (Fig. 2F). IRX5 overexpression also increased the expression of important adipogenic associated genes, such as *PPAR-γ, CEBP-α, LPL, FABP4* and *CD36* (Fig. 2H and Fig. S1G). Consistent with endogenous expression levels of IRX5, both Plvx/CTRL and Plvx/IRX5 groups exhibited growing *IRX5* levels during adipogenesis (Fig. 2G). On the other hand, knockdown of IRX5 was verified by qRT-PCR and western blot (Fig. 3A, B and Fig. S4). CEBP-α protein levels were down-regulated (Fig. 3B and Fig. S4). Oil red O staining confirmed a clear reduction for the accumulation of lipid droplets in IRX5 knockdown hMSCs (Fig. 3C); qRT-PCR analysis indicated that IRX5 knockdown inhibited the expression of *PPAR-γ*, *CEBP-α*, *CD36* and *FABP4* (Fig. 3D) after induction for 8 days. Thus, IRX5 overexpression promotes adipogenic differentiation of hMSCs, while IRX5 knockdown inhibits adipogenesis.

### Glycolysis pathway and PGC-1α are regulated by IRX5 overexpression

**Fig. 4:**
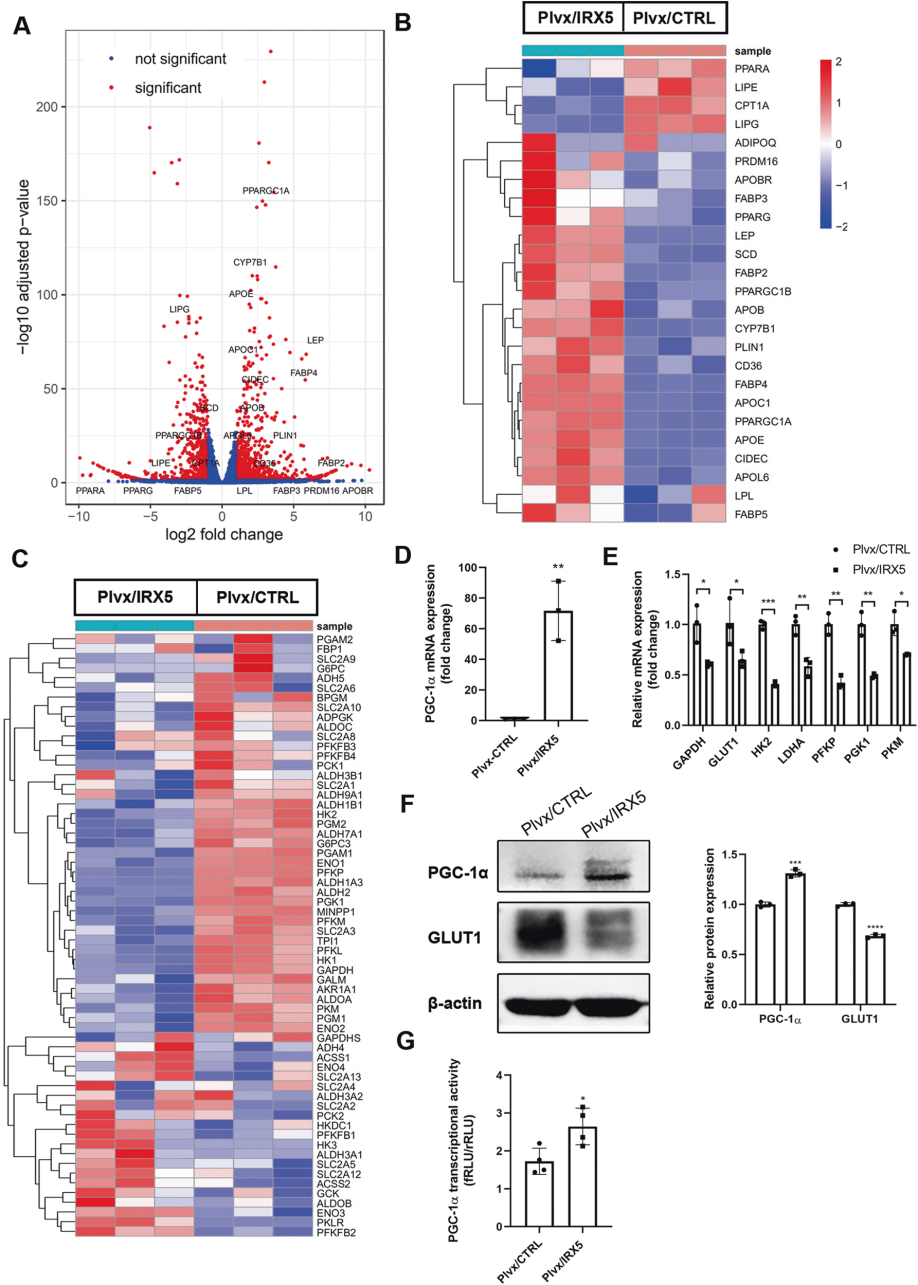
RNA sequencing analysis reveals the potential mechanism. **A** Volcano plot of differentially expressed genes (*n* = 3). **B** Heatmap plot of adipogenesis-associated genes (*n* = 3). **C** Heatmap of glycolysis-associated genes (*n* = 3). **D** qRT-PCR analysis of the mRNA level of *PGC-1α* in Plvx/CTRL and Plvx/IRX5 groups (*n* = 3). **E** qRT-PCR analysis of the mRNA level of *GAPDH*, *GLUT1*, *HK2, LDHA*, *PFKP*, *PGK1* and *PKM* in Plvx/CTRL and Plvx/IRX5 groups (*n* = 3). **F** Western blot analysis of the protein levels of PGC-1α and GLUT1 (*n* = 3). **G** Dual luciferase reporter assay shows that overexpression of IRX5 promotes the transcription activity of *PGC-1α* in 293E cells (*n* = 5). Results are shown as mean ± SD. * indicates a significant difference from the control group, *p < 0.05, ** *p* < 0.01, *** *p* < 0.001, ****p < 0.0001.

To gain a better understanding of the molecular mechanism of IRX5 in hMSCs, we determined global gene expression profiles by RNA sequencing (RNA-Seq). We analyzed genes which displayed significant differential expression identified by DEseq2 with adjusted *p*-value <0.05 and absolute log fold change >1. The volcano plot revealed that *PGC-1α* is one of the most significant genes changed in IRX5-overexpressing hMSCs (Fig. 4A). Heatmap analyses showed adipogenesis-associated genes were up-regulated in the Plvx/IRX5 group (Fig. 4B); whereas glycolysis-associated genes were down-regulated compared to control hMSCs (Fig. 4C). We tested and verified the results of RNA-seq through qRT-PCR and western blot. Consistently, overexpression of IRX5 promoted the expression of PGC-1α both in mRNA and protein levels significantly (Fig. 4D, F and Fig. S5); the major glycolysis-related genes were down-regulated by IRX5 overexpression as shown in qRT-PCR (Fig. 4E). Western blot showed that the protein expression of GLUT1, an important glucose transporter in the cell membrane, also decreased. These combined results suggest a reduced reliance on the glycolysis pathway (Fig. 4F and Fig. S5). In ST2 cells and HELA cells, we also found that PGC-1α were up-regulated and glycolysis were inhibited (Fig. S1C–F, S2C–E, S8B–C and S9B). Furthermore, we manually screened the potential promoter of PGC-1α for putative IRX5 binding sites. Human candidate PGC-1α promoter sequence from −1450 to +121 contains IRX5 binding sites (ACANNTGT). We then cloned the potential human PGC-1α promoter into the pGL3-basic luciferase reporter vector and performed a dual luciferase reporter assay. The results showed that IRX5 overexpression enhanced the transcriptional activity of PGC-1α (Fig. 4G), suggesting that PGC-1α is a possible pivotal transcriptional target of IRX5 in regulating adipogenic differentiation and energy metabolism.

**Fig. 5:**
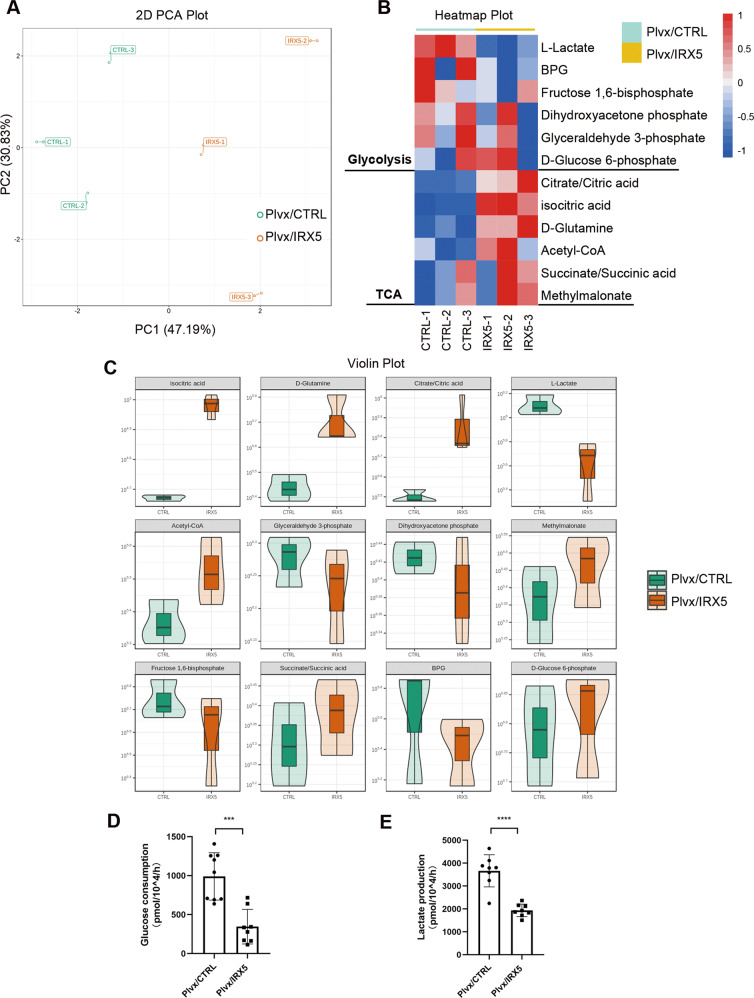
IRX5 overexpression inhibited glycolysis and promoted OxPhos. **A** Principal component analysis (PCA) (*n* = 3). Two-dimensional clustergram depicts the internal structure of the metabolomics data set with respect to variance (*n* = 3). **B** Heatmap of significantly changed metabolites in glycolysis and OxPhos pathway analyzed by LC-MS/MS (*n* = 3). **C** Violin plot compared the metabolites intensities in glycolysis and OxPhos pathway (*n* = 3). **D** Glucose uptake assay valued the rate of glucose consumption (*n* = 8). **E** Lactate production assay determined the rate of lactate production (*n* = 8). Results are shown as mean ± SD. *p < 0.05, ** *p* < 0.01, *** *p* < 0.001, ****p < 0.0001.

Energy-targeted metabolomics analysis was performed to investigate the function of IRX5 in energy metabolism. Cell deposition of Plvx/CTRL and Plvx/IRX5 groups was assayed by LC-MS/MS. 2D-Principal component analysis (PCA) plots of the metabolites varied significantly between Plvx/CTRL and Plvx/IRX5 groups (Fig. 5A), indicating that both groups have very different metabolic profiles. Heatmap and violin plots showed that some glycolytic metabolites, especially lactate, were reduced while levels of tricarboxylic acid cycle (TCA) metabolites were increased in IRX5 overexpressing hMSCs (Fig. 5B, C). Glucose uptake and lactate production assays demonstrated that the rate of glucose uptake and lactate production was lower in Plvx/IRX5 group (Fig. 5D, E). Taken together, these data indicated that IRX5 inhibits the glycolytic pathway and promotes TCA cycle.

### PGC-1α inhibitor and metformin activate glycolysis and rescue the excessive adipogenic differentiation of IRX5 overexpressing hMSCs

**Fig. 6:**
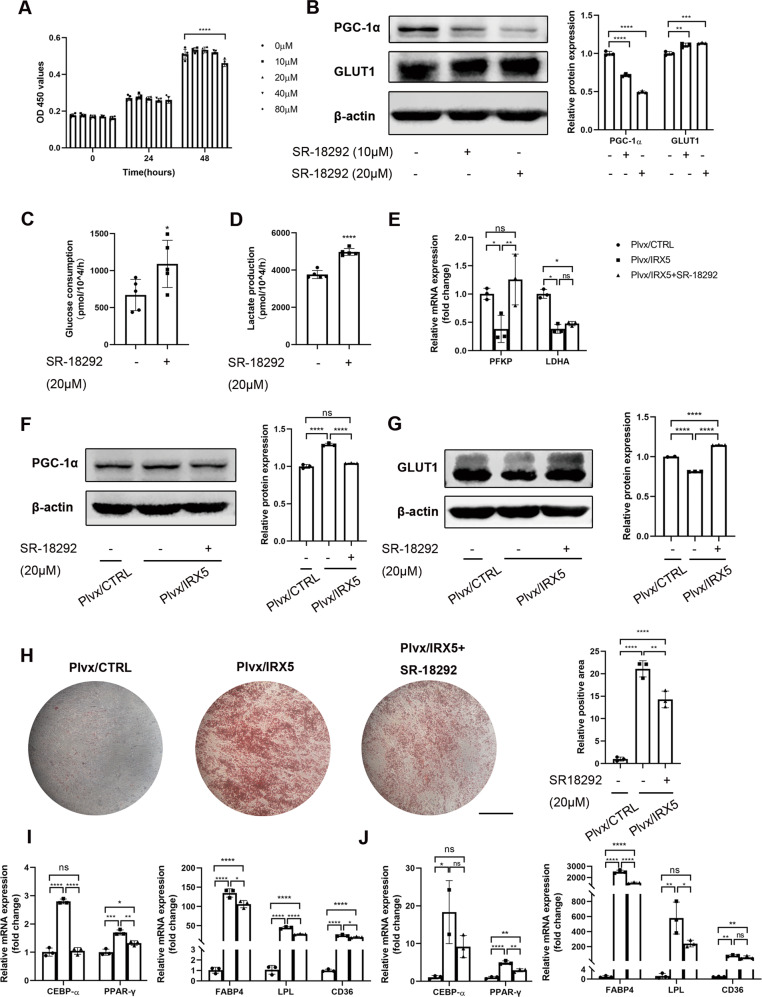
Inhibition of PGC-1α activated glycolysis and rescued the excessive adipogenic differentiation of IRX5 overexpressing hMSCs. **A** CCK-8 assay evaluated the toxicity of SR-18292, a selective inhibitor of PGC-1α (*n* = 5). **B** Western blot analysis of the levels of PGC-1α and GLUT1 in hMSCs after treatment with 10 μM and 20 μM of SR-18292 (*n* = 3). **C** Glucose uptake assay with treatment of 20 μM SR-18292 (*n* = 5). **D** Lactate production assay with treatment of 20 μM SR-18292 (*n* = 5). **E** qRT-PCR analysis of the levels of *PFKP* and *LDHA* in Plvx/CTRL, Plvx/IRX5 and Plvx/IRX5 treated with 20 μM of SR-18292 (Plvx/IRX5 + SR-18292) groups during adipogenic differentiation (*n* = 3). **F** Western blot analysis of the levels of PGC-1α in Plvx/CTRL, Plvx/IRX5 and Plvx/IRX5 + SR-18292 groups (*n* = 3). **G** Western blot analysis of the levels of GLUT1 in Plvx/CTRL, Plvx/IRX5 and Plvx/IRX5 + SR-18292 groups (*n* = 3). **H** Oil red O staining of Plvx/CTRL, Plvx/IRX5 and Plvx/IRX5 + SR-18292 groups at D16 after adipogenic induction (*n* = 3). **I** mRNA levels of *CEBP-α*, *PPAR-γ*, *FABP4*, *LPL* and *CD36* of Plvx/CTRL, Plvx/IRX5 and Plvx/IRX5 + SR-18292 groups at D4 after adipogenic induction (*n* = 3). **J** mRNA levels of *CEBP-α*, *PPAR-γ*, *FABP4*, *LPL* and *CD36* of Plvx/CTRL, Plvx/IRX5 and Plvx/IRX5 + SR-18292 groups at D8 after adipogenic induction (*n* = 3). Scale bar, 500 μm. Results are shown as mean ± SD. *p < 0.05, ** *p* < 0.01, *** *p* < 0.001, ****p < 0.0001.

To confirm the involvement of PGC-1α in the excessive adipogenic differentiation of IRX5 overexpressing hMSCs, we treated cells with a selective chemical inhibitor of PGC-1α, SR-18292, which has been shown to repress PGC-1α activation by increasing its level of acetylation [17]. The toxicity of SR-18292 was tested with CCK-8 assays, which showed that concentrations of SR-18292 lower than 80uM have no toxic effect on proliferation (Fig. 6A). We validated that 10 μM and 20 μM of SR-18292 significantly inhibited the protein level of PGC-1α and increased the protein level of GLUT1 (Fig. 6B and Fig. S6A). 20 μM of SR-18292 was chosen for the following experiments. SR-18292 increased the rate of glucose uptake and lactate production in hMSCs (Fig. 6C, D). Meanwhile, reduced mRNA expression of glycolysis-related genes, *PFKP* and *LDHA*, and GLUT1 protein expression in Plvx/IRX5 group were reversed by SR-18292 (Fig. 6E–G and Fig. S6B–C). Accumulation of lipid droplets of the Plvx/IRX5 group after 16 days of adipogenic induction was reduced by SR-18292 (Fig. 6H). qRT-PCR also revealed that *CEBP-α, PPAR-γ, FABP4, LPL* and *CD36* were down-regulated in the IRX5/Plvx upon SR-18292 treatment after 4 days (Figs. 6I) and 8 days (Fig. 6J) of adipogenic induction.

**Fig. 7:**
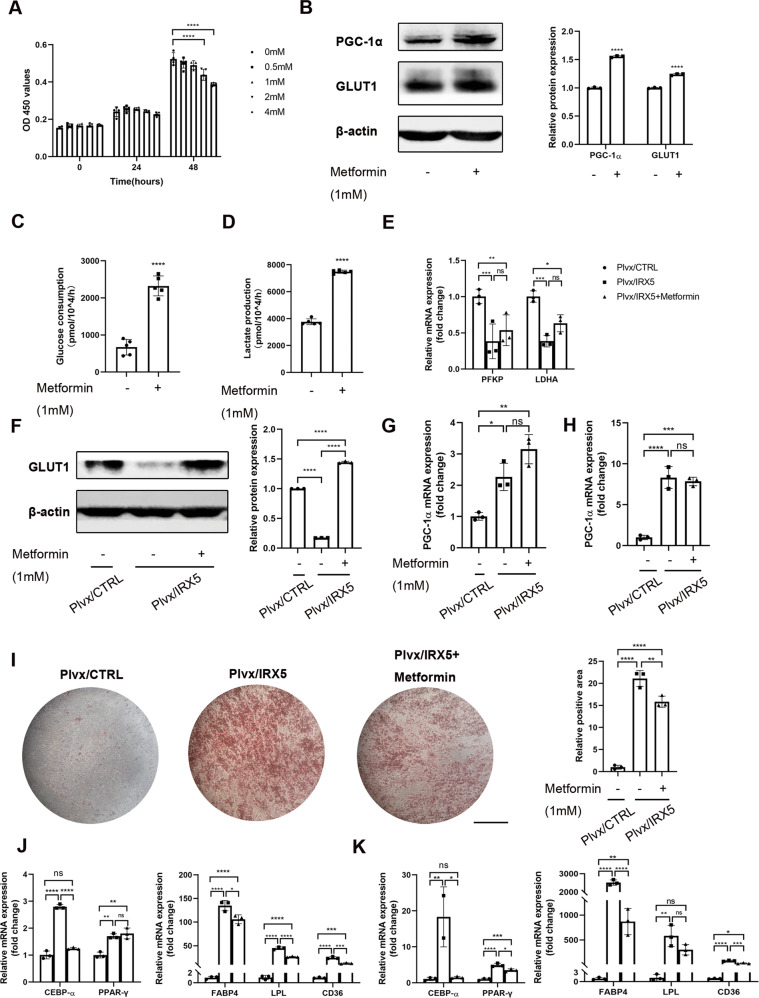
Activation of glycolysis rescued the excessive adipogenic differentiation of IRX5 overexpressing hMSCs. **A** CCK-8 assay evaluated the toxicity of metformin in hMSCs (*n* = 5). **B** Western blot analysis of the levels of PGC-1α and GLUT1 in hMSCs after treatment with 1 mM metformin (*n* = 3). **C** Glucose uptake assay was performed for the medium harvested from hMSCs after treatment with 1 mM metformin (*n* = 5). **D** Lactate production assay (*n* = 5). **E** qRT-PCR analysis of the levels of *PFKP* and *LDHA* in IRX5-control (Plvx/CTRL), IRX5-overexpressing (Plvx/IRX5) and IRX5-overexpressing treating with 1 mM metformin (Plvx/IRX5 + Metformin) groups during adipogenic differentiation (*n* = 3). **F** Western blot analysis of the levels of GLUT1 in Plvx/CTRL, Plvx/IRX5 and Plvx/IRX5 + Metformin groups (*n* = 3). **G** mRNA levels of *PGC-1α* in Plvx/CTRL, Plvx/IRX5 and Plvx/IRX5 + Metformin groups at D4 after adipogenic induction (*n* = 3). **H** mRNA levels of *PGC-1α* in Plvx/CTRL, Plvx/IRX5 and Plvx/IRX5 + Metformin groups at D8 after adipogenic induction (*n* = 3). **I** Oil red O staining of Plvx/CTRL, Plvx/IRX5 and Plvx/IRX5 + Metformin groups at D16 after adipogenic induction (*n* = 3). **J** mRNA levels of *CEBP-α*, *PPAR-γ*, *FABP4*, *LPL* and *CD36* in Plvx/CTRL, Plvx/IRX5 and Plvx/IRX5 + Metformin groups at D4 after adipogenic induction (*n* = 3). **K** mRNA levels of *CEBP-α*, *PPAR-γ*, *FABP4*, *LPL* and *CD36* in Plvx/CTRL, Plvx/IRX5 and Plvx/IRX5 + Metformin groups at D8 after adipogenic induction (*n* = 3). Scale bar, 500 μm. Results are shown as mean ± SD. *p < 0.05, ** *p* < 0.01, *** *p* < 0.001, ****p < 0.0001.

As a direct inhibitor of complex I in the electron transport chain (ETC) [18, 19], metformin has been used as an OxPhos inhibitor and glycolysis activator in many studies [20–22]. CCK-8 assays revealed that metformin did not affect the proliferation of hMSCs under concentrations of 1 mM (Fig. 7A). Levels of GLUT1 protein increased following treatment with 1 mM metformin for 24 hr (Fig. 7B and Fig. S7A). Activation of glycolysis was validated through glucose uptake and lactate production assay (Fig. 7C, D). mRNA levels of *PFKP* and *LDHA* were significantly higher in the Plvx/IRX5 group following metformin treatment (Fig. 7E). Using Western blot assays, we also found that GLUT1 protein expression was rescued by metformin (Fig. 7F and Fig. S7B). Protein levels of PGC-1α increased after metformin treatment in hMSCs (Fig. 7B and Fig. S7A), while mRNA expression of *PGC-1α* was only slighted affected by metformin in IRX5 overexpressing cells (Fig. 7G, H), indicating that glycolysis lies downstream of PGC-1α. Oil red O staining revealed that metformin reversed the increase of adipogenic differentiation in Plvx/IRX5 hMSCs (Fig. 7I). Moreover, mRNA levels of adipogenic-associated genes after 4 days (Fig. 7J) and 8 days (Fig. 7K) of adipogenic induction also revealed functional rescue of metformin. Overall, these findings demonstrate that IRX5 overexpression promotes adipogenic differentiation through PGC-1α regulation and glycolysis.

## Discussion

In this study, we demonstrated that IRX5 promotes adipogenesis of hMSCs by increasing the expression of PGC-1α and inhibiting glycolysis. These changes can be reversed with PGC-1α inhibitor and metformin.

Marrow adipose tissue (MAT) accumulates naturally with age and in metabolic diseases like obesity [23]. Understanding the lineage commitment of BMSCs is critical for understanding bone homeostasis and for improvements in regenerative medicine. Adipogenesis of hMSCs involves two steps: commitment to adipogenic lineage cells followed by maturation into a functional adipocyte. Adipogenesis of hMSCs was induced with an induction cocktail containing l-methyl-3-isobutylxanthine (IBMX), dexamethasone, insulin, and Rosiglitazone [24]; maintained in an insulin-only medium. The accumulation of lipid-rich vacuoles within cells was readily visible once stained red with Oil Red O solution. We first verified osteogenic and adipogenic differentiation of hMSCs, which displayed classical differentiation patterns, indicating that hMSCs can be used as an appropriate cell model for investigating cell differentiation.

In the current study, we found that expression of IRX5 was upregulated during adipogenic induction. Overexpression of IRX5 in hMSCs promotes the expression of adipogenic-related genes and significantly improves the accumulation of lipid droplets. The pro-adipogenic effect of IRX5 is consistent with the study on the peripheral adipose tissues which showed that adult IRX5 knock-out mice had reduced fat mass and were protected from high fat diet-induced fat accumulation [25]. Large-scale clinical research studies demonstrated that a variant of FTO enhances the expression of IRX5 and IRX3 leading to excessive adipocyte differentiation and obesity [15, 26, 27]. However, this is contrary to one mouse study which showed that loss of Irx3 and Irx5 increases the hypertrophic chondrocytes transition toward adipocytes, and that the amount of marrow fat tissue in the neonatal distal tibia increases in these animals [28]. It has been revealed that there are two types of adipocytes in BM. Constitutive MAT (cMAT) develops early on in the distal skeletal region, contains large adipocytes resembling WAT, and is relatively devoid of active hematopoiesis; Regulated MAT (rMAT) containing single adipocytes interspersed throughout is colocalized with active hematopoiesis in proximal skeletal regions [29]. Based on the description in this study [28], more cMAT are accumulated to the distal tibia (cMAT) in Irx3-null or Irx5-null animals. While it is still unknown if rMAT and cMAT share the same progenitors, the lipid composition and gene expression are definitely different. This may help to explain previous inconsistency in the relationship between IRX5 and different adipose tissues. Two mutations in human IRX5 cause a recessive congenital disorder, Hamamy syndrome, resulting in skeletal abnormalities, osteopenia and severe cardiac defects [30]. The phenotype present in humans is not observed in Irx5^−/−^ mice, suggesting that the function of IRX5 in humans is distinct from its function in mice [30–35]. Furthermore, the osteopenia in Hamamy Syndrome couldn’t be explained by the promoting adipogenic effect revealed in our study. However, accumulating evidence shows that the inverse correlation between MAT and bone mass is not always causally linked. For example, a subset of patients with congenital generalized lipodystrophy 1 (CGL1) or CGL2 (who lack MAT) develop osteolytic cyst-like lesions in the long bones [36]. Therefore, it is important to dissect the role of IRX5 on differentiation using hMSCs cell model.

Glucose, a major source of ATP for mammalian cells, can be metabolized by oxidative phosphorylation (OxPhos) and/or glycolysis [37]. BMSCs predominantly reside in regions of the BM cavity, which provides a hypoxic niche with the oxygen concentration of 1%-6% [38]. Under this condition, BMSCs tend to produce energy through glycolysis, reflecting their adaptation to low oxygen levels and their relatively low energy demand [39, 40]. The rewiring of glucose metabolism has also been implicated in cell lineage commitment [37]. For example, the Wnt/LRP5 pathway could promote osteogenic differentiation of mouse BMSCs through glycolysis [41], and notch signaling is known to partially suppress osteoblast differentiation by restricting glycolysis [42]. Additionally, the rate of glycolysis and increased formation of OxPhos supercomplexes seem to be characteristic of hMSC adipogenic differentiation [43–48]. Thus, adipogenesis of hMSCs is closely associated with changes in glucose metabolism. The data in this study indicate that IRX5 promotes PGC-1α expression and inhibits glycolysis. PGC-1α is a transcriptional co-activator of PPAR-γ and acts as a key regulator of mitochondrial biogenesis and oxidative metabolism in many tissues [49–51]. Some studies have revealed that PGC-1α modulates adipogenic differentiation [46, 52–54]. Acetylation of PGC-1α, which can be induced by SR-18292 [17], is used as a means of controlling its transcriptional activity and metabolic function. Metformin at suprapharmacological concentration could inhibit mitochondrial respiration, thereby compensatorily boosting glycolysis to produce ATP [55]. Here, we show that IRX5 overexpression in hMSCs led to increased PGC-1α expression and reduced glycolysis. The increased adipogenesis in hMSCs overexpressing IRX5 could be partially reversed by SR-18292 and metformin. Together, these observations implicate PGC-1α as an upstream driver of glycolysis and adipogenesis. Importantly, glycolysis is also a driver for adipogenesis, rather than a consequence of increased PGC-1α.

Energy metabolism during cell commitment is a multifaceted process. Therefore, understanding cell adaptation during differentiation and its molecular regulation could shed light on the application and therapy of BMSCs. Overall, the data presented in this manuscript suggest that hMSCs with IRX5 overexpression may shift their energy production preference from glycolysis to OxPhos, which is beneficial for adipogenesis.

**Fig. 8:**
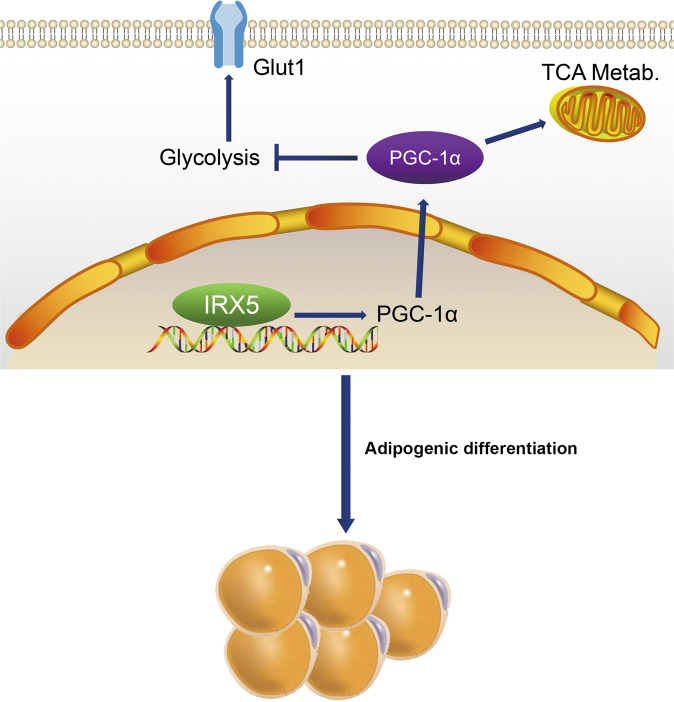
Schematic diagram of the proposed role of IRX5 in the regulation of adipogenic differentiation of hMSCs. Our results suggested that IRX5 promotes adipogenic differentiation of hMSCs through targeting PGC-1α and restraining glycolysis. SR-18292, PGC-1α inhibitor, and metformin, potential glycolysis booster, reversed the effect of IRX5 overexpression on adipogenesis.

In conclusion, IRX5 is accompanied by the adipogeneis of hMSCs; this adipogenesis is increased by IRX5 overexpression. PGC-1α inhibition and glycolysis activation rescue IRX5 modulated adipogenesis (Fig. 8). Moreover, further in-depth study of the relationship between IRX5 and bone homeostasis under physical and pathological conditions, as well as the underlying regulatory mechanism, are needed.

## Materials and methods

### Cell cultures and reagents

Human bone marrow-derived mesenchymal stem cells (hMSCs) were purchased from ScienCell (CA, USA) and cultured in mesenchymal stem cell medium (MSCM; ScienCell). hMSCs were incubated at 37 °C in a humidified atmosphere of 5% CO_2_ and were used at passage 3–7. SR-18292 (PGC-1α inhibitor) was purchased from Cayman (Michigan, USA) and metformin was purchased from Selleck (Texas, USA). Adipogenic differentiation induction medium and adipogenic differentiation maintenance medium were used for adipogenic induction (Cyagen, Guangdong, China).

### Adipogenic differentiation

hMSCs were seeded in 6 well plate or 24 well plate at 5 × 10^4^ cells/cm^2^. When the cells reached 100% confluence, the growth medium was replaced with an induction medium for 3 days and then a maintenance medium for an additional day. The cycle of induction and maintenance was repeated at least three times for optimal induction. After the cells differentiated, they were fixed with 4% paraformaldehyde for 10 min and stained with oil red O solution (Cyagen) for over 2 h. The lipid droplets were observed and photographed by an inverted microscope (Leica, Hessen, Germany).

### Osteogenic differentiation

When the cells reached 70%-80% confluence, osteogenic differentiation medium, containing 10 mM β-glycerophosphate (Sigma-Aldrich, MO, USA), 50 μg/mL ascorbic acid (Sigma-Aldrich), and 1% Pen/Strep (Hyclone, UT, USA), was added and changed every day. ALP activity assay and ALP staining were conducted after osteogenic differentiation for 7 days as previously described [56].

After osteogenic induction for 21 days, mineral nodules were detected by staining with alizarin red S (pH=4.2; Servicebio, Hubei, China) for 1 hr.

### Lentivirus production and transduction

The lentivirus vector (pGLV8/ mCherry/ Puro) overexpressing human or mouse IRX5 gene and vector (pGLV10/ mCherry/ Puro) containing shRNAs targeting IRX5 were packaged and purified by GenePharma (Shanghai, China). hMSCs, 293E cells, HELA cells and ST2 cells were infected with lentivirus in the presence of 5 μg/mL polybrene (Sigma-Aldrich) for 12–24 h. 48 h later, the infection efficiency was observed by fluorescent microscopy. Puromycin (2ug/ml) was used to select infected cells. For brevity, Cells with IRX5-overexpressing/empty lentivirus and IRX5-shRNA/Ctrl lentivirus infection were referred as Plvx/IRX5, Plvx/CTRL, IRX5-shRNA, and Ctrl-shRNA, respectively.

### CCK-8 assay

hMSCs were seeded into 96-well plates with 10,000 cells for each well and the cell viability was tested as previously described [57].

### qRT-PCR

Total RNA was extracted from cultured cells using a Trizol reagent kit (CWBIO, Beijing, China). The concentration of total RNA was measured by NanoDrop 2000 (Thermo Fisher., MA, USA). cDNA was transcribed from 1 μg of RNA using PrimeScript^TM^ Reverse Transcription Reagent Kit (Takara Bio, Kyoto, Japan). qRT-PCR was performed with TB Green Premix Ex Taq II Kit (Takara Bio) using QuantStudio 6 (Applied Biosystems, MA, USA). The primer sequences are listed in Table S1.

### Western blot analysis

Proteins were collected on ice using RIPA (Beyotime, Shanghai, China) with phosphatase inhibitor (Roche Applied Science, Baden-Württemberg, Germany) and protease inhibitor (PMSF; Roche Applied Science). The concentration of protein was measured with a BCA kit (Biosharp, Anhui, China). 30 μg of protein were electrophoresed on 8%-10% SDS-polyacrylamide gel and transferred onto methanol-treated PVDF membrane (Millipore, MA, USA). The membranes were blocked with 5% non-fat milk for over 1 h and incubated with anti-IRX5 (Sigma-Aldrich, SAB1404807, MO, USA), anti-PGC-1α (Santa Cruz, SC-517380, Texas, USA), anti-GLUT1 (Abcam, ab115730, Cambridge, UK), anti-PPAR-γ (Abcam, ab178806, Cambridge, UK), anti-CEBP-α (Cell Signaling Technology, D56F10, Cambridge, UK), anti-β-actin (BioPM, PMK081W, Hubei, China), anti-β-Tubulin (Abmart, M20005F, Shanghai, China) antibodies at 4 °C overnight. An ECL system (Millipore) was used to visualize the target bands. Image J software (National Institutes of Health, Maryland, USA) was used for quantification by densitometry.

### Transcriptome analysis

An RNeasy mini kit (Qiagen, Düsseldorf, Germany) was used to isolate total RNA from hMSCs. Library construction and sequencing were performed at Shanghai Sinomics Corporation. An Average of 49 million reads per sample were obtained. Trim Galore was applied for automatic quality and adapter trimming (https://www.bioinformatics.babraham.ac.uk/projects/trim_galore). The processed reads were aligned to the human reference genome HG38 by HISAT2 [58]. As a result, an average alignment rate of 85% was obtained. Sample read counts were summarized by StringTie2 [59] with the GENCODE V32 annotation file. Differential expression analysis was determined based on read counts by using DESeq2 [60]. P-values of DESeq2 were corrected using the Benjamini-Hochberg [61] procedure for multiple testing adjustment. The genes were ranked by the Wald statistics reported by DESeq2 followed by the pre-ranked GO term GSEA analysis [62].

### Dual luciferase assay

Potential *PGC-1α* promoter (-1450bp to +121 bp) was amplified and then cloned into pGL3-basic vectors as reporter plasmids (See Table S2 for *PGC-1α* promoter sequences). 293E cells transfected stably with blank lentivirus or IRX5-overexpressing lentivirus were established and seeded on 24-well plates at a density of 1 × 10^5^ cells per well. Then, the plasmids were transfected as previously described [57] and *PGC-1α* transcriptional activity (the light units of firefly luciferase/Renilla luciferase, fRLU/rRLU) was calculated.

### LC–MS/MS assay

#### Cell collection

3 × 10^6^ cells were seeded in 75 cm^2^ culture flasks and cultured until the cells reached over 90% confluence. Cells were washed with cold PBS twice, harvested by cell scrapers, and centrifuged at 1000 g for 10 min at 4 °C. The supernatant was discarded and the deposition was quick-frozen in liquid nitrogen and kept at −80 °C.

#### Sample preparation

Samples were thawed on ice. 500 μl pre-cooled 80% methanol aqueous solution was added and then whirled for 2 min. The mixture was frozen for 5 min in liquid nitrogen and thawed again. The thaw-frozen steps were repeated 3 times. Once the samples were centrifuged at 15,000 rpm at 4 °C for 20 min, the supernatant was transferred into the sample bottles for LC–MS/MS analysis.

### Glucose uptake assay and lactate production assay

Cells were seeded on a 6 well plate at a density of 5 × 10^5^ cells per well. After 24 h, the medium was replaced with no phenol red medium. After incubation for 24 h, the medium was collected, and the cell numbers were counted.

The glucose uptake assay and lactate production assay were performed as previously described [63].

### Statistical analysis

All experiments were repeated in triplicate. The data were analyzed through Graphpad Prism Version 8.0. Results were expressed as mean ± SD and comparisons were analyzed by Student’s *t* test for two groups and ANOVA for more than two groups. *P* values < 0.05 were considered statistically significant.

## Supplementary information

Supplementary Information

Figure S1

Figure S2

Original Western Blots

Table S1

Table S2

## Data Availability

The RNA-seq raw data have been deposited in the GEO database. Geo number is GSE195679.
