# Supplementary material for: IRX5 promotes adipogenesis of hMSCs by repressing glycolysis
Source: Cell Death Discov. 2022 Apr 15;8:204. doi: 10.1038/s41420-022-00986-7 (PMC9012830; doi:10.1038/s41420-022-00986-7)
Supplement: Supplementary file 1 — Supplementary Information [file 41420_2022_986_MOESM1_ESM.docx]

**Supplementary Figure Legends**

**Figure S1 Effect of IRX5 in ST2 cells**

A and B, IRX5 overexpression efficiency were determined by qRT-PCR (A) and Western blot (B). C, qRT-PCR showed mRNA levels of *Pgc-1α* were up-regulated by Irx5 (n=3). D, Western blot revealed that protein levels of PGC-1α were up-regulated in Plvx/IRX5 group. E, qRT-PCR showed that mRNA of *Glut1* and *Hk2* were decreased in Plvx/IRX5 group (n=3). F, Western blot showed that protein levels of GLUT1 were also down-regulated by IRX5 overexpression. G, ST2 cells were cultured in adipogenic differentiation medium, finding that at D0, D5 and D15, adipogenic associated genes such as *Fabp4* and *Lpl* were up-regulated in Plvx/IRX5 group (n=3). Results are shown as mean ± SD. * indicates a significant difference from the control group, *p<0.05, ** p<0.01, *** p<0.001, ****p<0.0001.

**Figure S2 Functions of IRX5 in HELA cells**

A and B, IRX5 overexpression efficiency were determined by qRT-PCR (A) and Western blot (B). C, qRT-PCR analysis of the mRNA level of *PGC-1α* in Plvx/CTRL and Plvx/IRX5 groups (n=3). D, Western blot analysis of the protein levels of PGC-1α. E, qRT-PCR analysis of the mRNA level of *PFKP* and *LDHA* in Plvx/CTRL and Plvx/IRX5 group (n=3). Results are shown as mean ± SD. * indicates a significant difference from the control group, *p<0.05, ** p<0.01, *** p<0.001, ****p<0.0001.

**Figure S3 Uncropped images of blots presented in main Figure 2.**

**Figure S4 Uncropped images of blots presented in main Figure 3.**

**Figure S5 Uncropped images of blots presented in main Figure 4.**

**Figure S6 Uncropped images of blots presented in main Figure 6.**

**Figure S7 Uncropped images of blots presented in main Figure 7.**

**Figure S8 Uncropped images of blots presented in supplementary Figure 1.**

**Figure S9 Uncropped images of blots presented in supplementary Figure 2.**

**Supplementary Table Legends**

**Table S1 Primer sequences for qRT-PCR.**

**Table S2 Promoter sequences of PGC-1α.**
