# Supplementary figures and images for: IRX5 promotes adipogenesis of hMSCs by repressing glycolysis
Source: Cell Death Discov. 2022 Apr 15;8:204. doi: 10.1038/s41420-022-00986-7 (PMC9012830; doi:10.1038/s41420-022-00986-7)

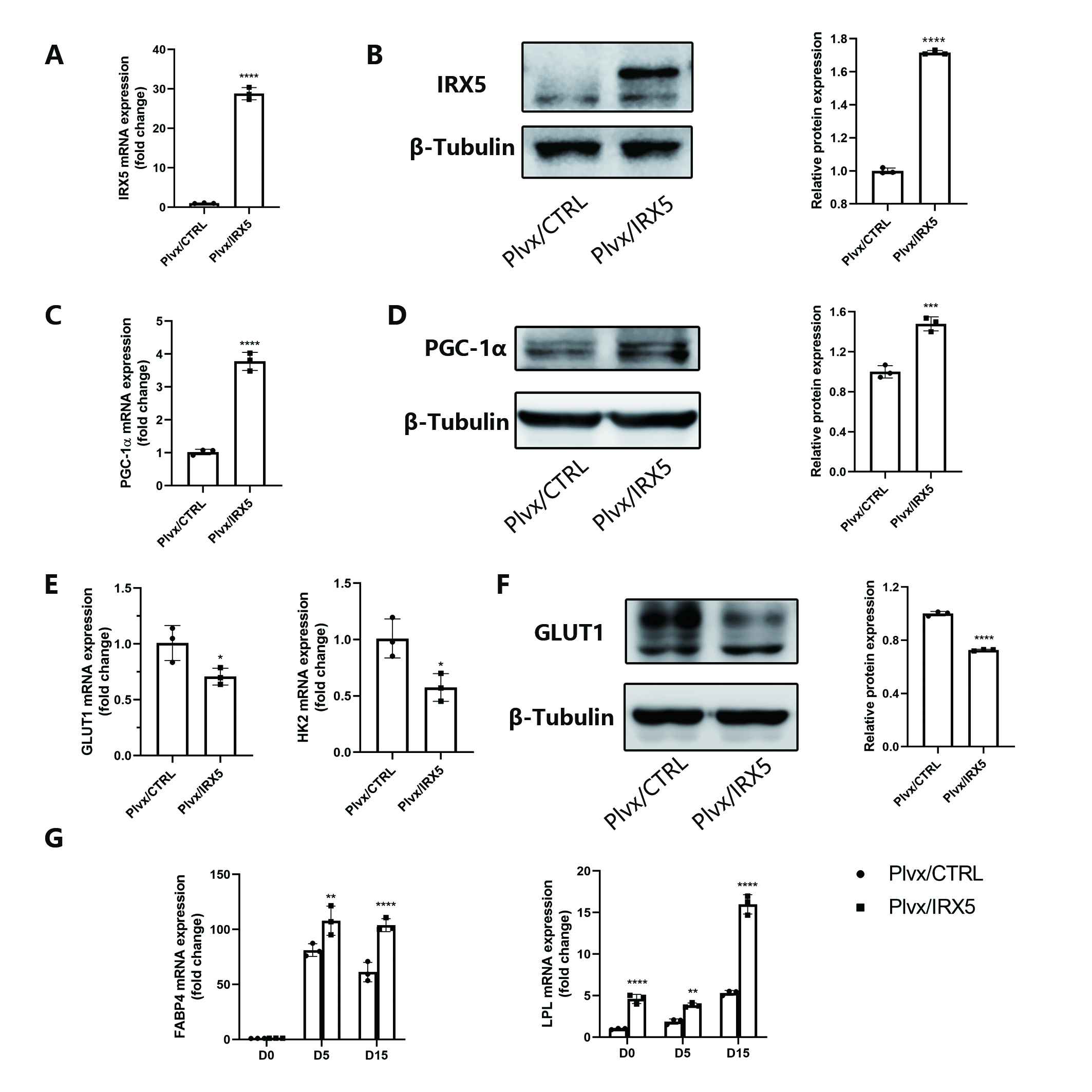

Supplement: Supplementary file 2 — Figure S1 [file 41420_2022_986_MOESM2_ESM.tif]

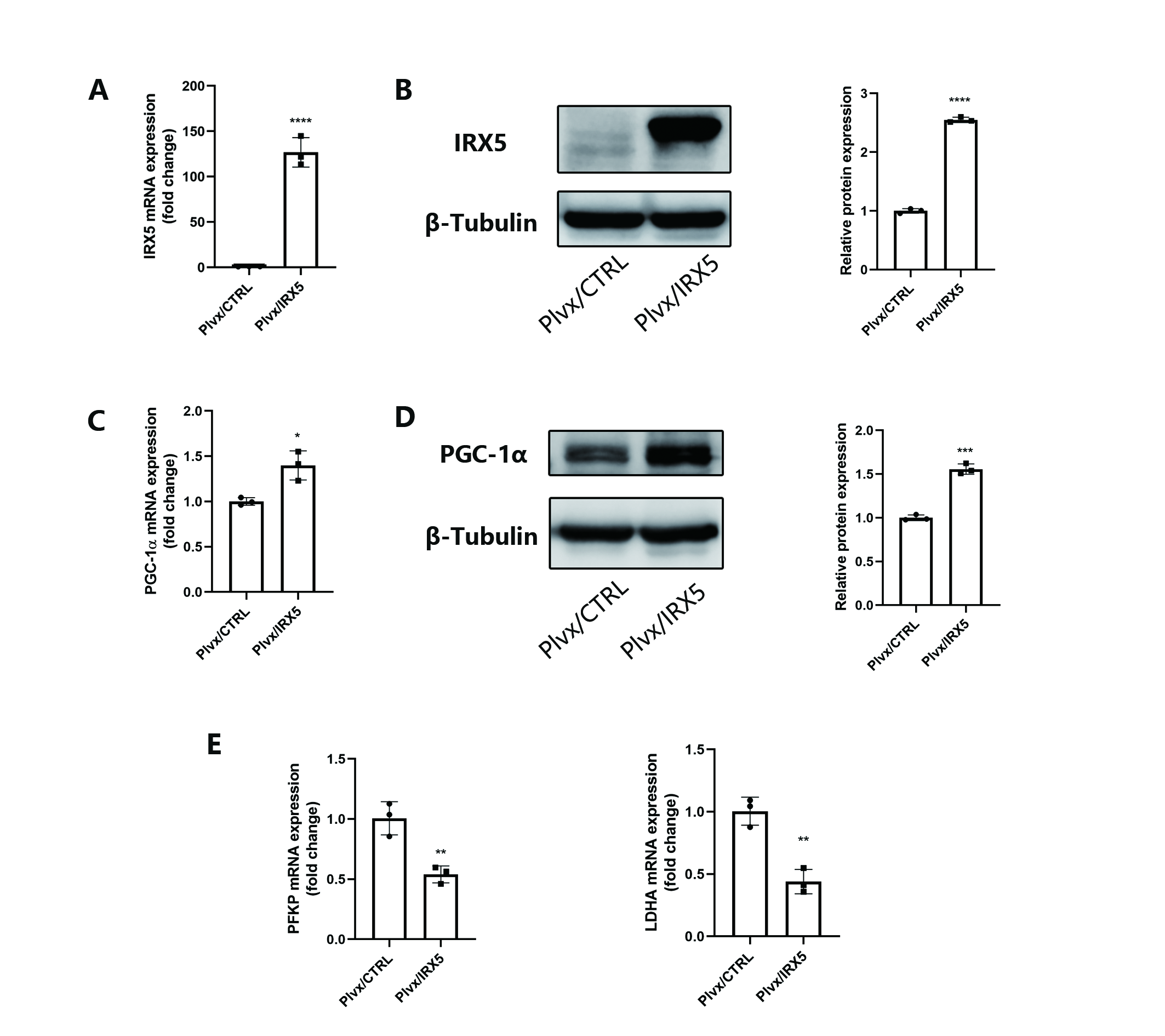

Supplement: Supplementary file 3 — Figure S2 [file 41420_2022_986_MOESM3_ESM.tif]
