# Supplementary material for: IRX5 promotes adipogenesis of hMSCs by repressing glycolysis
Source: Cell Death Discov. 2022 Apr 15;8:204. doi: 10.1038/s41420-022-00986-7 (PMC9012830; doi:10.1038/s41420-022-00986-7)
Supplement: Supplementary file 4 — Original Western Blots [file 41420_2022_986_MOESM4_ESM.pdf]

Figure S3

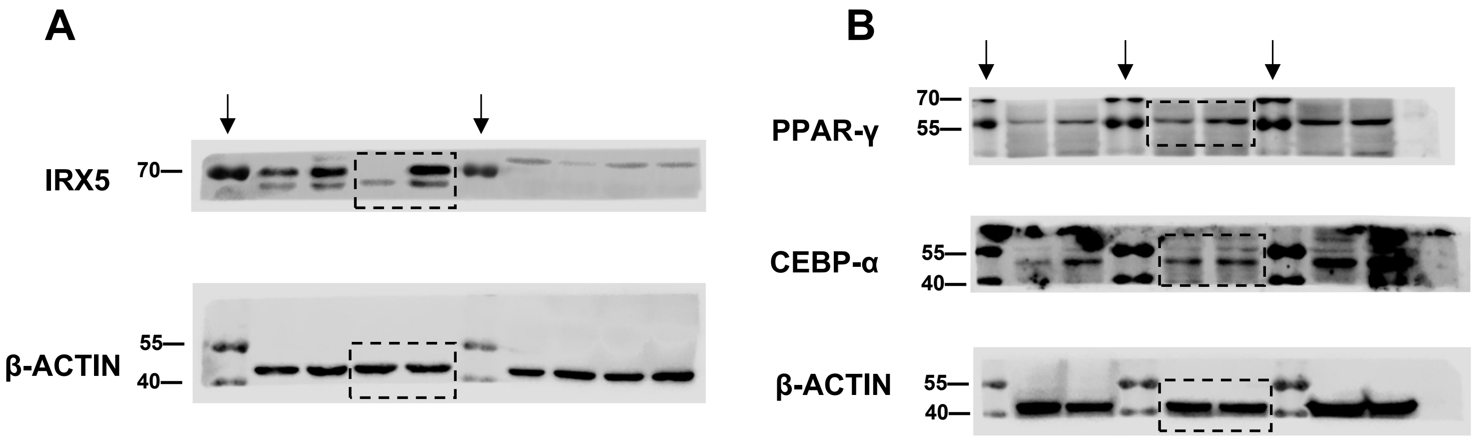

Uncropped images of blots presented in main Figure 2 (Black arrows indicate protein ladders)

Figure S4

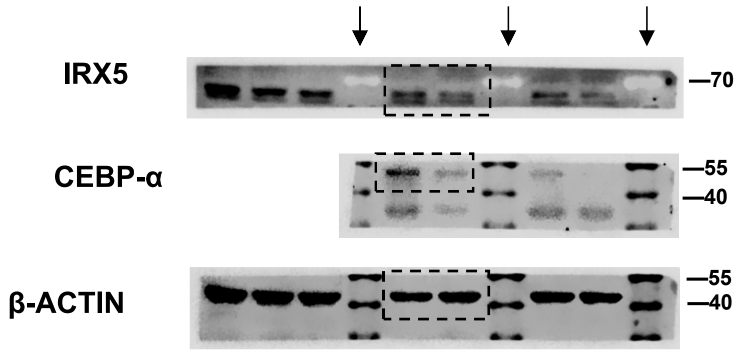

Uncropped images of blots presented in main Figure 3

Figure S5

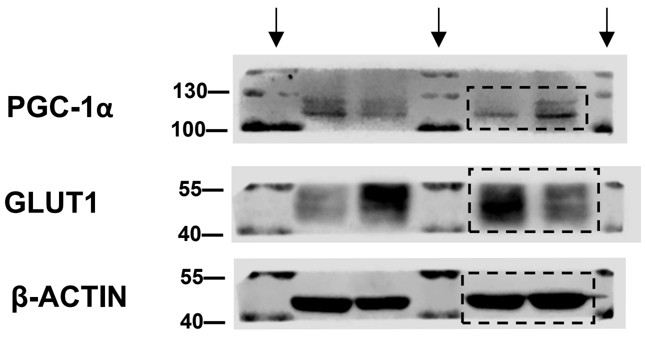

Uncropped images of blots presented in main Figure 4

Figure S6

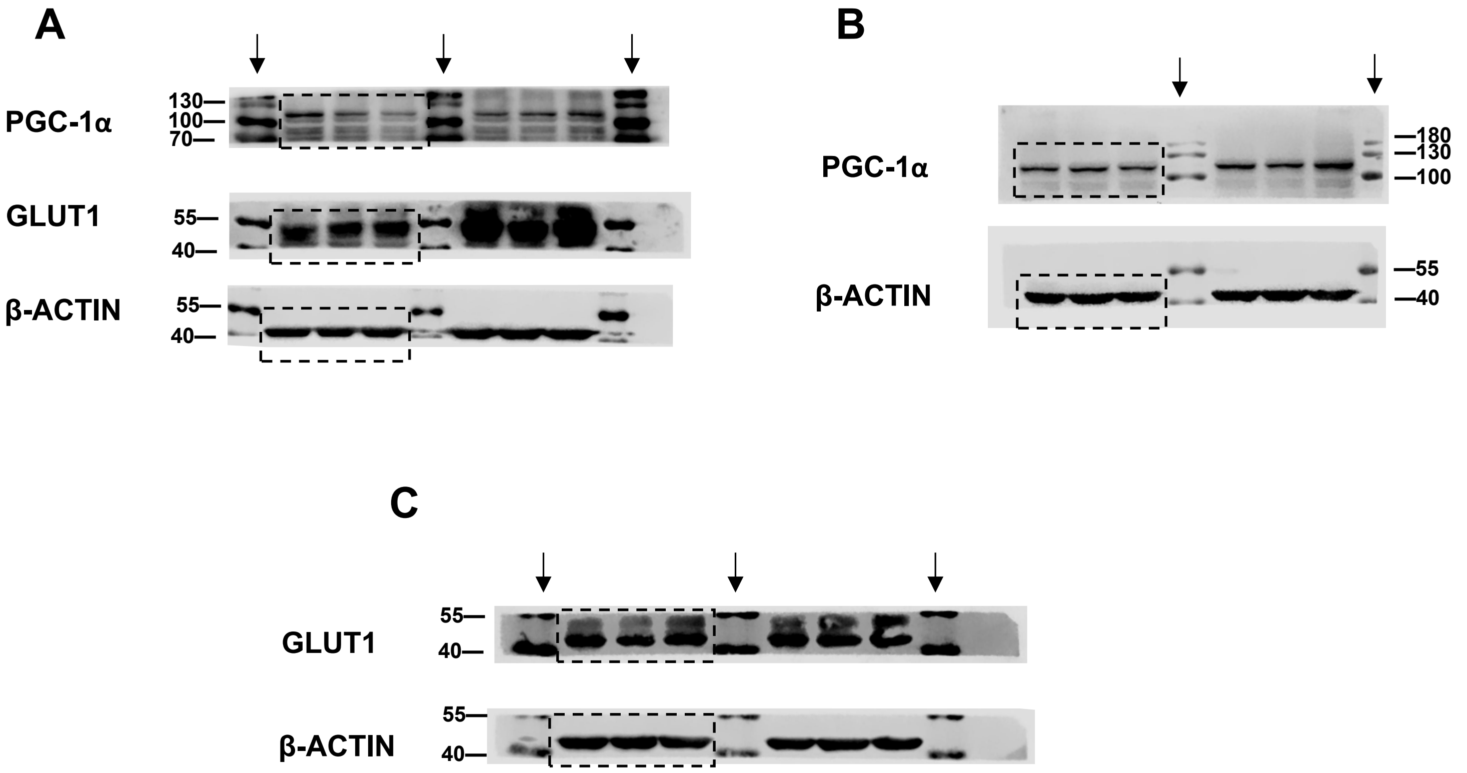

Uncropped images of blots presented in main Figure 6

Figure S7

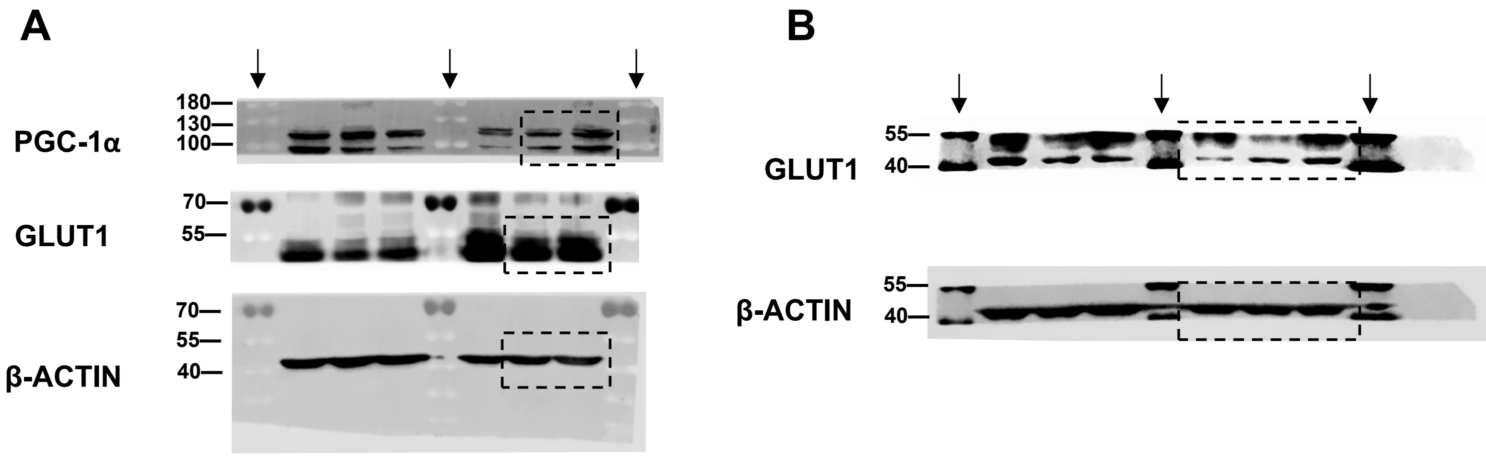

Uncropped images of blots presented in main Figure 7

Figure S8

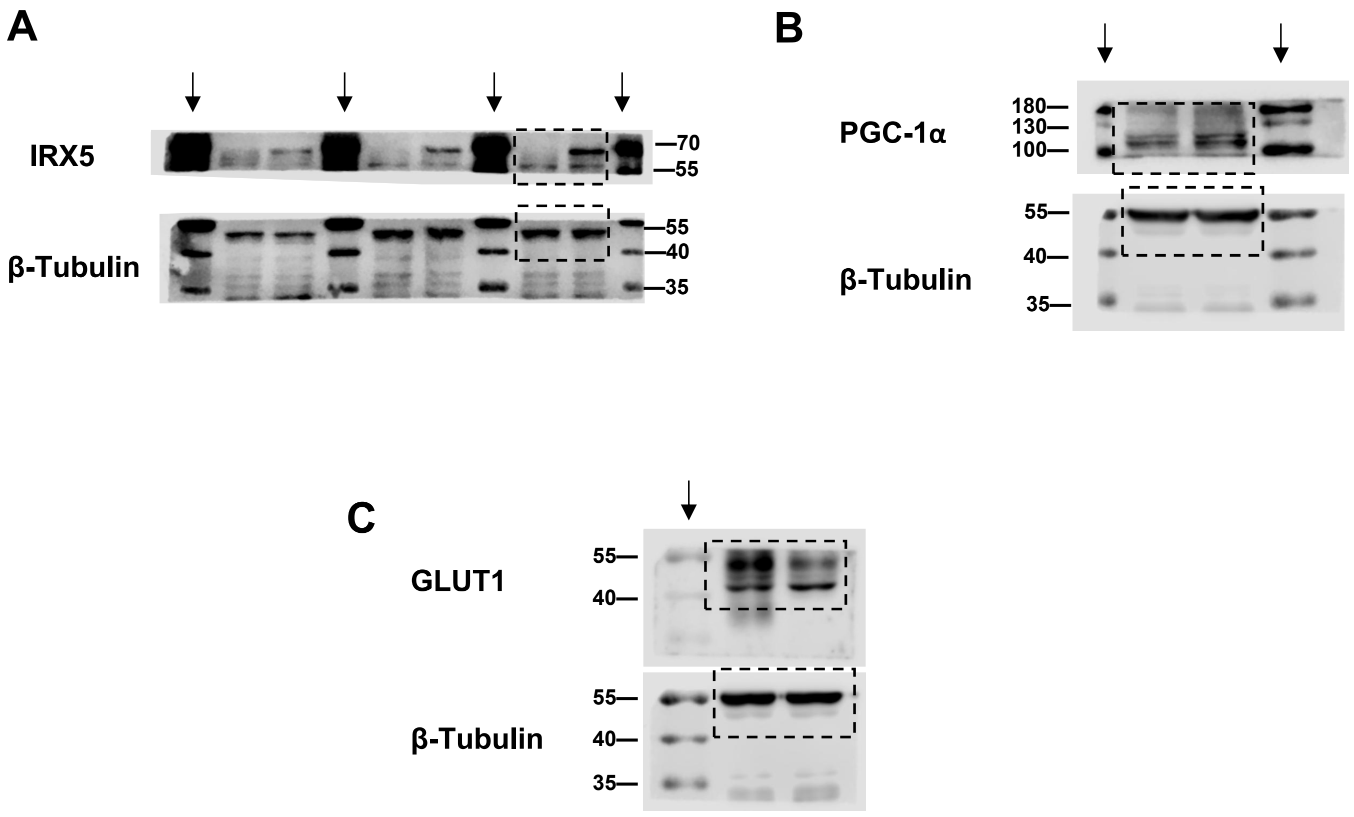

Uncropped images of blots presented in supplementary Figure 1

Figure S9

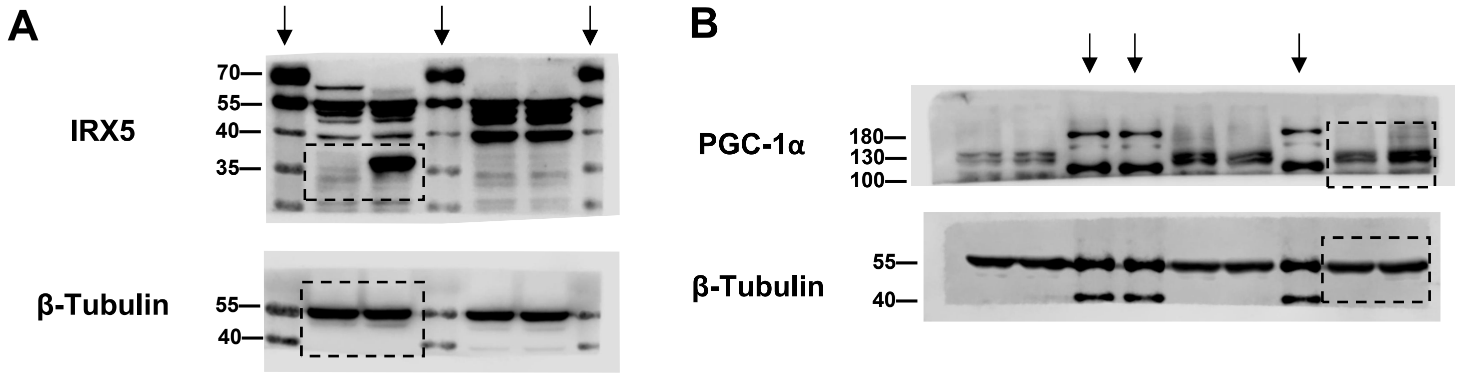

Uncropped images of blots presented in supplementary Figure 2
