# Supplementary material for: IRX5 promotes adipogenesis of hMSCs by repressing glycolysis
Source: Cell Death Discov. 2022 Apr 15;8:204. doi: 10.1038/s41420-022-00986-7 (PMC9012830; doi:10.1038/s41420-022-00986-7)
Supplement: Supplementary file 5 — Table S1 [file 41420_2022_986_MOESM5_ESM.docx]

**Table S1 Primer sequences for qRT-PCR.**

**Homo sapiens (human)**

| Gene | Forward primer (5'-3') | Reverse primer (5'-3') |
| --- | --- | --- |
| IRX5  PGC-1α  RPL13A | GCAAAGACTCTCCCTATGA  ACGCACCGAAATTCTCCCTT  ACAAGTTGAAGTACCTGGCTT | CTCTCTCGGTGATGGAAA  GGCGCTCTTCAATTGCCTTC  TTTTGTGGGGCAGCATACCT |
| PPAR-γ | GCCGTGGCCGCAGATTTGAA | TGGTGGGCCAGAATGGCATC |
| CEBP-α | TGCCTAGGAACACGAAGCACG | TGGGGCACAGAGGCCAGATA |
| FABP4 | CCTTAGATGGGGGTGTCCTGGT | GCCTTTCATGACGCATTCCACC |
| CD36 | TTGCAAAACGGCTGCAGGTC | CATCACCAATGGTCCCAGTCTCAT |
| LPL | GCCCCGAGATGGAGAGCAAA | TCTCTTCTTTGGTCGGCGGC |
| RUNX2 | ACCACAAGTGCGGTGCAAAC | ACTGCTTGCAGCCTTAAATGACTCT |
| IBSP | CAGGCCACGATATTATCTTTACA | CTCCTCTTCTTCCTCCTCCTC |
| COL1A1 | GGATTCCAGTTCGAGTATGG | CAGTGGTAGGTGATGTTCTG |
| ALPL | GAGTATGAGAGTGACGAGAAAG | GAAGTGGGAGTGCTTGTATC |
| SPP1 | CCCTGACCCATCTCAGAAGC | TGGTCATGGCTTTCGTTGGA |
| GAPDH | TGTTCGACAGTCAGCCGCAT | CGCCCAATACGACCAAATCCGT |
| GLUT1 | ATGGCCGGGGTCCTATAAAC | GCTCGCTGTTGCTACCTCTT |
| PFKP | AGAGATCGCCACACAGATGC | CTCCAGGAGTCCCAGGTAGG |
| HK2 | AAATGGAGCGAGGTCTGAGC | CCCACTTCCCATTCCGAACA |
| PKM | ACCATGCGGAGACCATCAAG | GTAGGCGTTATCCAGCGTGA |
| PGK1 | CCACTGTGGCTTCTGGCATA | ATGAGAGCTTTGGTTCCCCG |
| LDHA | ACTGCAAACTCCAAGCTGGT | TTCACGTTACGCTGGACCAA |

**Mus musculus (house mouse)**

| Gene | Forward primer (5'-3') | Reverse primer (5'-3') |
| --- | --- | --- |
| Irx5  Pgc-1α  Rpl13a | CGCCTCAAGAAAGAGAACAAGATGGTTGCCTGCATGAGTGTGTG  GAGGTCCTCAAGACCAACGG | CTGCAGCCGTTCGCA  AGATCTGGGCAAAGAGGCTG  GTGCGCTGTCAGCTCTCTAA |
| Hk2 | CTGCTTTGGAGATCCGAGGG | GTCTAGCTGCTTAGCGTCCC |
| Glut1 | GAGTGACGATCTGAGCTACGG | CTCCCACAGCCAACATGAGG |
| Fabp4 | GGATTTGGTCACCATCCGGT | TTCCATCCCACTTCTGCACC |
| Lpl | GGAGAAGCCATCCGTGTGAT | CTCAGGCAGAGCCCTTTCTC |
