# Supplementary material for: IRX5 promotes adipogenesis of hMSCs by repressing glycolysis
Source: Cell Death Discov. 2022 Apr 15;8:204. doi: 10.1038/s41420-022-00986-7 (PMC9012830; doi:10.1038/s41420-022-00986-7)
Supplement: Supplementary file 6 — Table S2 [file 41420_2022_986_MOESM6_ESM.docx]

**Table S2. Promoter sequences of PGC-1α**

TTCTTTCACTTTAATATTTGAGCAATTTAATATTTAAAATATGTGTTAGGGCAAATAACCATGTTTTTCTGTTTAAGGAGATGGACAATGAAGAACAGTGTAAATTAACCTCAGTAGAATAATGCTTTACAAATTATATTTAGGTAACTGGGGGATTGTTTTCAGGTAAATTATTATCAAGTAAAAGAATTGAGCTGATTCTAAAAGTATATTGGGTTTAACATTTTATGAAAACATGTTTATTCACACAGATACATACACGTGTACATCTCCTGGGAACTTGGAAGGGTTAAGTCAGACCACTGTCATGTTACAGGAAGTGTTTTTCCACTGTGTCCAGTACCTTGAGTTTGTTATGTATTCAATATGTAGTGTCATCATAAAACAGTTGCACCTACCTGCATTAGCCCTCATTGTCTCAAGGTACAAGCTGAAAAATAATAGAAAGTAAAGCTCAGGAATTGAATATTTCTGCTAATAGTGTGTTGGTATTTTTCCCTCAGTTCACAGACATTCTTGATTTCAAAACGCAAACTACACAACCCAGGGCACTAGGGTTGGAATTCAATGTTTATTCAAAAAGGCACCCTAAGGCAGTTAGGGAGGAAACGCTACATGTATGAAAAATAGGAGCCGGGAATCAAAGCTGATCTGAGCAGAGCAGCAGCGACTGTATTTACTAACACTTGTTTTCTGGGAGCCTATGAGAGAAATGGAAATAATTAGAAGGAAGCTGAAAGGATGGGGTTTTGTGGCTTGTTCTCCTTATATGGAGCAAAGAAAACTGCAGCAACTCTTCGGGAGCTGGTATTCCCTACTGCCATGGGGGCAGCCGAATTCTGGGTGGAGGAGTTTGTTTATACCTTAACACATACAGGCTATTTTGTTGATTAAACAAGCAAAAAAAAAAAAAAAAAAAAAAAAAGCCCCGTTTGCGCTTTCAAACACTCCCTCAATGAGAAAATGTCTCATAAAAATGCATCATGTGATAAGCTCTTGCTTTAGTCCCAAACTGAGCTTGAGTCCACTTGGAGATCTTAGAATTAAAGAGTTCTTAGGGAATACACGTTTTAGCTAAGAATATAGTTACTCTGTCATGAAACAGGGAGCTTTGCCACTTGCTTGTTTTGGAAGGAAAATAAATTAAAAAAAGATTGCAGGGGATTTTGGTTATTATATGGCCAGGGCTCCGTTTAGAGTCTGTGGCATTCAAAGCTGGCTTTAATCACAGCATGATGCTTGAAGCCTCCAAAAGTCTAAGTGTTTCCTTTCTTTCTTTCTTTTCTTTTTCTTTCTTTTTTTTTTTTTTTAAAGCGTTACTTCACTGAAGCAGAGGGCTGCCTTTGAGTGACGTCACGAGTTAGAGCAGCAAGCTGCACAGGGGAAGGGAGGCTGGGTGAGTGACAGCCCAGCCTACTTTTTAATAGCTTTGTCATGTGACTGGGGACTGTAGTAAGACAGGTGCCTTCAGTTCACTCTCAGTAAGGGGCTGGTTGCCTGCATGAGTGTGTGCTCTGTGTCACTGTGGATTGGAGTTGAAAAAGCTTGACTGGCGTCATTCAGGAGCTGGAT
